# Spatial analysis of positive and negative Q fever laboratory results for identifying high- and low-risk areas of infection in the Netherlands

**DOI:** 10.3402/iee.v3i0.20432

**Published:** 2013-11-28

**Authors:** Elsa J. van den Berg, Cornelia C. H. Wielders, Peter M. Schneeberger, Marjolijn C. Wegdam-Blans, Wim van der Hoek

**Affiliations:** 1 Centre for Infectious Disease Control, National Institute for Public Health and the Environment, Bilthoven, The Netherlands; 2 Department of Medical Microbiology and Infection Control, Jeroen Bosch Hospital, 's-Hertogenbosch, The Netherlands; 3 Department of Medical Microbiology, Laboratory for Pathology and Medical Microbiology (PAMM), Veldhoven, The Netherlands

**Keywords:** zoonosis, *Coxiella burnetii*, goats, the Netherlands, case–control, risk factors, epidemiology

## Abstract

**Background:**

The Netherlands faced a large Q fever epidemic from 2007 to 2010, in which thousands of people were tested for the presence of antibodies against *Coxiella burnetii* as part of individual patient diagnosis. So far, only data of notified cases were used for the identification of high-risk areas, which can lead to misclassification of risk. Therefore, we identified high- and low-risk areas based on laboratory test results to make control measures more efficient.

**Methods:**

Data on diagnostic Q fever laboratory tests were obtained from two regional laboratories of medical microbiology in the high-incidence area in the south of the Netherlands. The proportion of patients testing positive was mapped per postal code area. Patients testing positive were compared to patients testing negative based on the distance between residential address and the nearest infected goat farm with adjustment for age and sex.

**Results and conclusion:**

Of 11,035 patients tested, 4,011 (36.4%) had a positive laboratory test result for Q fever. Maps showing the spatial pattern of tests performed and proportion of positive tests allowed for the identification of high- and low-risk Q fever areas. The proportion of patients testing positive was higher in areas close to infected goat farms compared to areas further away. Patients living <1 km from an infected goat farm had a substantially higher risk of testing positive for antibodies to *C. burnetii* than those living >10 km away (OR 21.70, 95% CI 16.28–28.92). Laboratory test results have the potential to make control measures more efficient by identifying high-risk areas as well as low-risk areas.

Q fever is a zoonosis caused by the intracellular bacterium *Coxiella burnetii*. This bacterium has a worldwide distribution in different animal species, but sheep and goats are considered the main reservoir (1). Especially when infected pregnant small ruminants abort, billions of *C. burnetii* are introduced into the environment (2). The bacterium has the ability to persist in the environment, and therefore, even weeks to months after the birthing event there can be a risk for infection. Transmission to humans mainly occurs through inhalation of contaminated aerosols and dust particles (3). Among persons with clinical symptoms, there is usually an acute onset with fever, headache, fatigue, and frequently an atypical pneumonia or hepatitis. However, acute Q fever is reported to be asymptomatic in 50–60% of the cases (4).

Since 1975, notification of patients with acute Q fever (i.e. patients presenting with fever or pneumonia or hepatitis and with a positive Q fever laboratory result) is mandatory in the Netherlands. From 2007 to 2010, the southern part of the Netherlands faced large seasonal outbreaks of acute Q fever, with the highest peak in 2009 (5–7). During the epidemic, more than 4,000 human cases were notified, which makes it the largest epidemic of Q fever reported worldwide (8). Before the first outbreak, acute Q fever was notified sporadically with 1–32 Dutch human cases annually (6).

The majority of infections occurred in the eastern part of the province of Noord-Brabant, where a clear epidemiological link was established with dairy goat farms that experienced high Q fever-induced abortion rates (9). Based on human notification data, higher attack rates were found in areas around such farms compared to areas further away (9). In contrast to goats, most sheep are kept on non-dairy (meat) sheep farms and these did not seem to play an important role during the Q fever epidemic (10). Veterinary control measures, implemented after the peak in 2009, have therefore primarily focused on dairy goat farms. Since these actions, which included mandatory mass vaccination, culling of pregnant animals on infected farms, and hygiene measures, incidence has declined (8, 11).

Monitoring of the human epidemic was entirely based upon notification data. This allowed for the identification of high-incidence areas (10). However, notification data are of limited use in studying risk factors, because a control group is generally not available for comparison. Without a control group, areas could be misclassified as low risk when Q fever patients are not tested, and therefore not notified. Similarly, other areas with active case finding could be misclassified as high risk. When the nationwide mandatory bulk tank milk monitoring started in October 2009, all households situated within a radius of 5 km of a positive farm were informed through an official letter. This might have influenced peoples’ health-seeking behaviour and diagnostic testing practices of physicians. Therefore, in the present study, we compared geographic data of patients testing positive with those testing negative in Q fever laboratory tests. Identification of low-risk areas, in addition to high-risk areas would allow for more focused and therefore more cost-efficient control measures.

## Methods

### Study design

In this geographical case–control study, data were gathered and combined from two regional laboratories located in the high-incidence Q fever area. The geographic region was limited to two-digit postal code regions, where at least 500 people were tested for Q fever. Postal codes in the Netherlands consist of four numbers followed by two uppercase letters. The first two digits indicate a city and a region; the full six-digit postal code generally represents part of a street. Within the study area, four-digit postal code areas were selected as the spatial unit of analysis, as this is the smallest unit in the Netherlands for which reliable routine data are available. The total number of four-digit postal code areas in the Netherlands is 4,005, with a median area size of 5.3 km^2^ (range 0.1–132 km^2^). Population size for each four-digit postal code area in 2010 was obtained from Statistics Netherlands (12).

### Laboratory data

Anonymous data on serum samples tested for Q fever were obtained from two laboratories located in the east of the province of Noord-Brabant ('s-Hertogenbosch and Veldhoven). In the laboratories, sera were analysed to detect the presence of IgG and IgM antibodies against phases I and II of *C. burnetii* using different techniques. In this study, an IgG phase II titre of ≥1:32 measured by immunofluorescence assay (IFA; Focus Diagnostics) was considered positive. When a serum was tested with the complement fixation test (CFT; Virion/Serion), a phase II titre >1:4 was considered positive. A fourfold IgG II titre rise or more was defined as confirmed acute Q fever for both serological tests. In persons for whom only a *C. burnetii* PCR (polymerase chain reaction) was performed, the PCR was decisive for the outcome. IgM results were not used in the analysis.

We included positive test results over the period January 1, 2009 – December 31, 2010. Negative test results were included for the period January 1, 2010–December 31, 2010 only, because persons testing negative in 2009 could have become positive in 2010. For people testing negative in 2010, we assumed that they must have been negative in 2009 as well, since IgG antibodies are detectable for a long time after infection (13). Besides Q fever laboratory test outcome, information on age, sex, and postal code were available for analysis. We did not use laboratory tests that were done as part of scientific (seroprevalence) studies. Seroprevalence studies were done among patients with risk factors for chronic Q fever and among people with occupational exposure. The analysis was therefore limited to tests that were requested by physicians for individual patient diagnosis. We only included one test result per person, whereby a patient with negative and positive test results was classified as ‘positive’.

### Animal data

Locations of small ruminants (goats and sheep) were available from the national registration system of the Ministry of Economic Affairs for November 2009. Information on small ruminant farms that experienced abortion waves (defined as >5% abortions of all pregnant animals) caused by *C. burnetii* was provided by the Animal Health Service. Locations of bulk tank milk positive farms were available at the website of the Food and Consumer Product Safety Authority (14). Infected farms included in the analysis were notified between 2005 and 2010.

### Data analysis

The analysis focused on the high-incidence area in the south of the Netherlands. Locations of farms and residence locations of all persons tested for Q fever in this area were geo-referenced to *X*- and *Y*-coordinates based on the centroid of their full postal code (generally part of a street). Distance between home address of the persons tested and the nearest infected farm was calculated. Incidence and proportion of patients tested and patients testing positive were mapped to identify high- and low-risk areas. We defined ‘risk’ as the probability of a positive test result in a postal code area, in different categories.

Patients with positive test results were compared to those with negative test results based on shortest distance between an infected farm and the home address. We used the Chi-square test to compare proportions. Student's *t*-test and Mann–Whitney *U* test were used to compare the continuous variables, that is, age and distance. Logistic regression was used for risk factor analysis in which explanatory variables were categorised, depending on the distribution of the variable. For each outcome, the strength of its association with a positive laboratory test outcome was expressed in odds ratios (OR) with 95% confidence interval (95% CI).

Statistical analysis was performed using SAS (version 9.3; SAS Institute, Cary, NC). ArcGIS (ArcGIS 9.3.1, Esri, Redlands, CA) was used to calculate the distance between residential address and the nearest farm and to create maps.

## Results

**Fig. 1:**
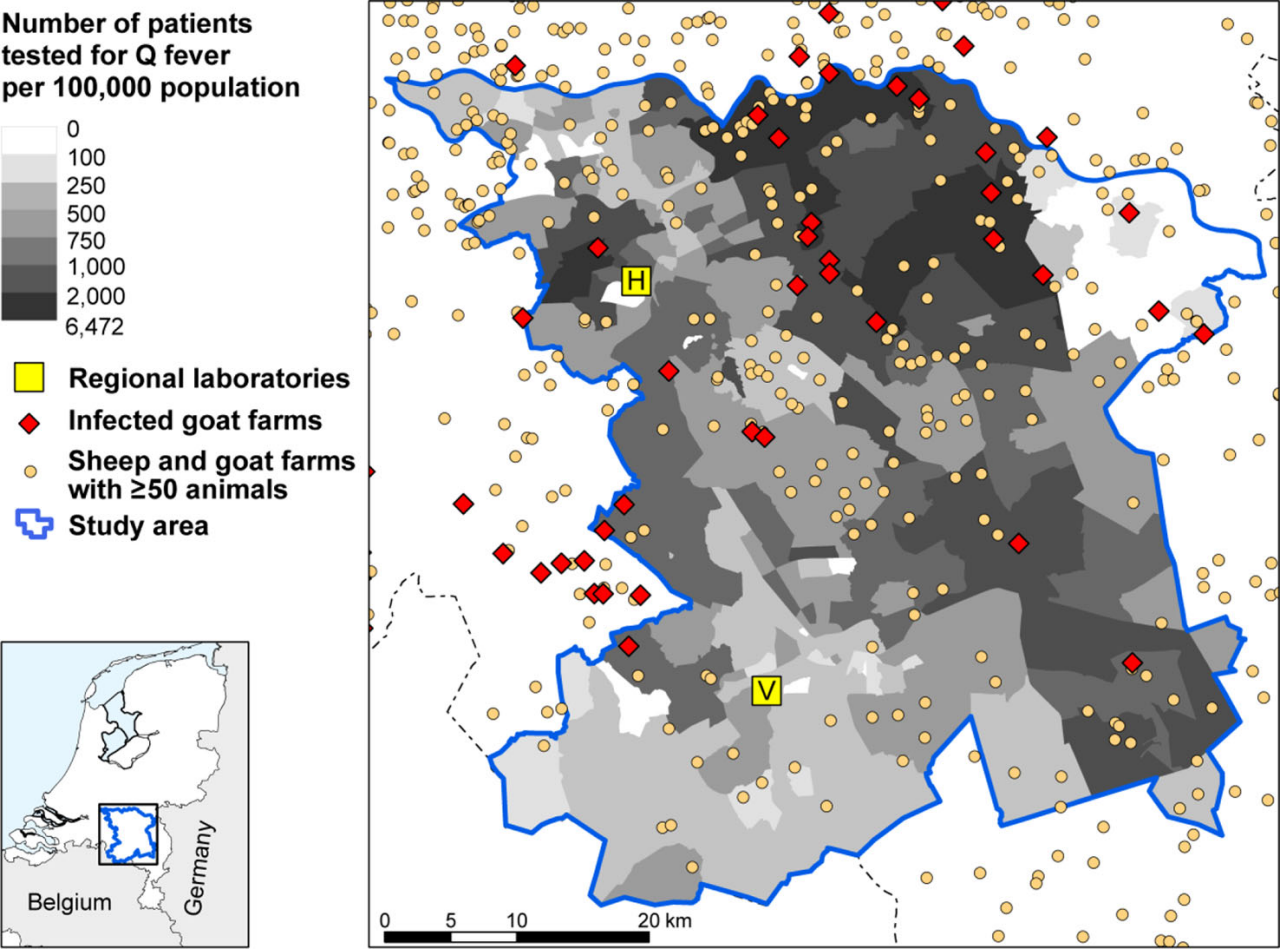
Incidence (×100,000 population) of Q fever laboratory tests (n =11,035) with locations of all small ruminant farms with ≥50 animals in the south of the Netherlands by four-digit postal code area. Data from patients testing positive in 2009 and 2010, and patients testing negative in 2010 at the laboratories in 's-Hertogenbosch (H) and Veldhoven (V).

**Fig. 2:**
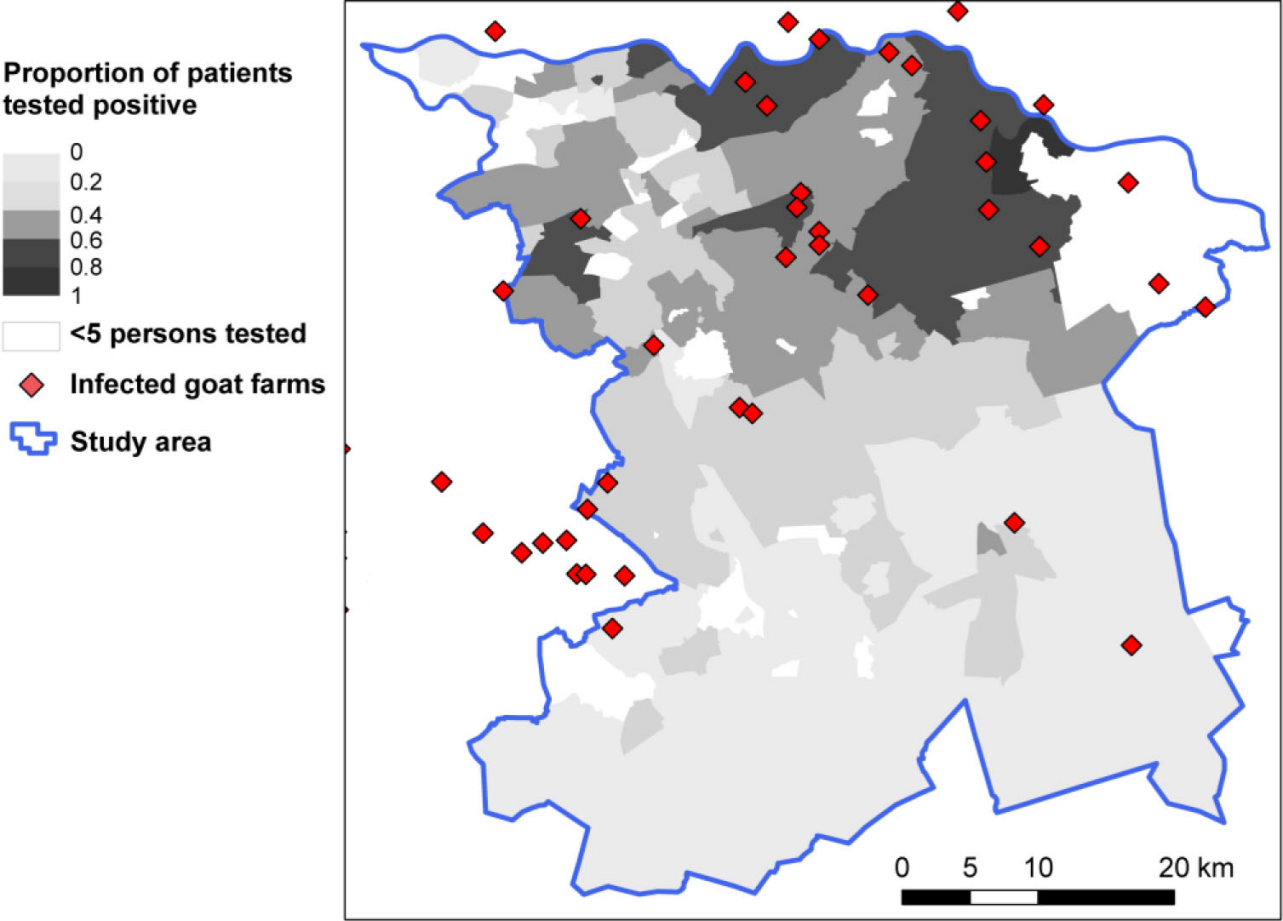
Proportion of positive test results based on the same data as in Fig. 1 with locations of infected goat farms.

The study area consisted of 262 four-digit postal code areas, with 1,324,790 inhabitants in total. In the selected area, 11,035 persons were tested and included in the analysis. The incidence of patients tested was highest in the east of the selected study area (Fig. 1). In the northern part of the study area, there are postal code areas where many persons were tested, of which the majority had a positive result (Fig. 2). In contrast, in some areas of the southern part where many persons were tested, there was a lower proportion of patients testing positive. Areas where fewer than five tests were performed, which were classified as areas with unknown risk, are indicated in Fig. 2.

There were 460 small ruminant farms with ≥50 animals per farm in the study area, the large majority being sheep farms (Fig. 1). Infection status is only known for the 90 milk-producing farms that were subjected to the strictly enforced bulk tank milk monitoring program. The milk-producing farms included one dairy sheep farm, which remained negative in the monitoring program, and 89 dairy goat farms, of which 41 testing positive or experienced Q fever-induced abortion waves.

In total, 4,011 (36.4%) patients had a positive test result for Q fever. They were more often male and were younger than the patients with a negative test result (Table 1). On average, the patients testing positive were living 4.1 km from an infected dairy goat farm compared with 6.7 km for the patients testing negative. In logistic regression analysis, with positive or negative laboratory result as outcome measure, distance was divided in seven categories with patients living >10 km from the nearest infected goat farm as reference (Table 2). Individuals living <1 km from an infected goat farm had a much higher risk of testing positive for antibodies to *C. burnetii* than those living >10 km away (OR 21.70, 95% CI 16.28–28.92). The greater the distance from residential address to the nearest infected goat farm, the lower the risk for a positive laboratory test for Q fever. Adult age (20 ≤ 60 years) and male sex remained significant risk factors for a positive test result in the multivariate analyses.

**Table 1:** Characteristics of individuals with a positive or negative laboratory test result

	All	Positive		Negative		*p*
				
Variables	*N*	*N*	%	*N*	%	
Sex						
Male	5,507	2,224	55.4	3,283	46.7	*p*<0.001*
Female	5,528	1,787	44.6	3,741	53.3	
	Mean	Mean	IQR	Mean	IQR	
Age (years)	50.1	48.3	38–60	51.1	37–67	*p*<0.001**
Distance (km)^a^	5.7	4.1	2.1–5.4	6.7	3.7–9.3	*p*<0.001***
All	11,035	4,011	36.4	7,024	63.7	

IQR, interquartile range.

a Distance between patient's home address and nearest infected dairy goat farm.

* Chi-square test

** Student's *t*-test

*** Mann–Whitney *U* test.

**Table 2:** Multivariate logistic regression analysis of risk factors associated with testing positive for Q fever

	All	Positive	Positive		
			
Variables	*N*	*N*	%	Odds ratio	95% Confidence interval
Age (years)					
0 ≤ 20	1,000	245	24.5	Reference	
20 ≤ 60	6,236	2,722	43.7	2.71	2.31–3.19
60 ≤ 100	3,799	1,044	27.5	1.42	1.20–1.69
Sex					
Male	5,507	2,224	40.4	1.52	1.40–1.66
Female	5,528	1,787	32.3	Reference	
Distance^a^ (km)					
< 1 km	345	229	66.4	21.70	16.28–28.92
1 ≤ 2 km	1,058	682	64.5	19.73	15.90–24.47
2 ≤ 4 km	2,682	1,162	43.3	8.16	6.76–9.86
4 ≤ 6 km	3,346	1,286	38.4	6.62	5.50–7.98
6 ≤ 8 km	1,260	405	32.1	5.14	4.17–6.34
8 ≤ 10 km	633	104	16.4	2.06	1.57–2.71
≥ 10 km	1,711	143	8.4	Reference	
Total	11,035	4,011	36.4		

a Distance between patient's home address and nearest infected dairy goat farm.

**Fig. 3:**
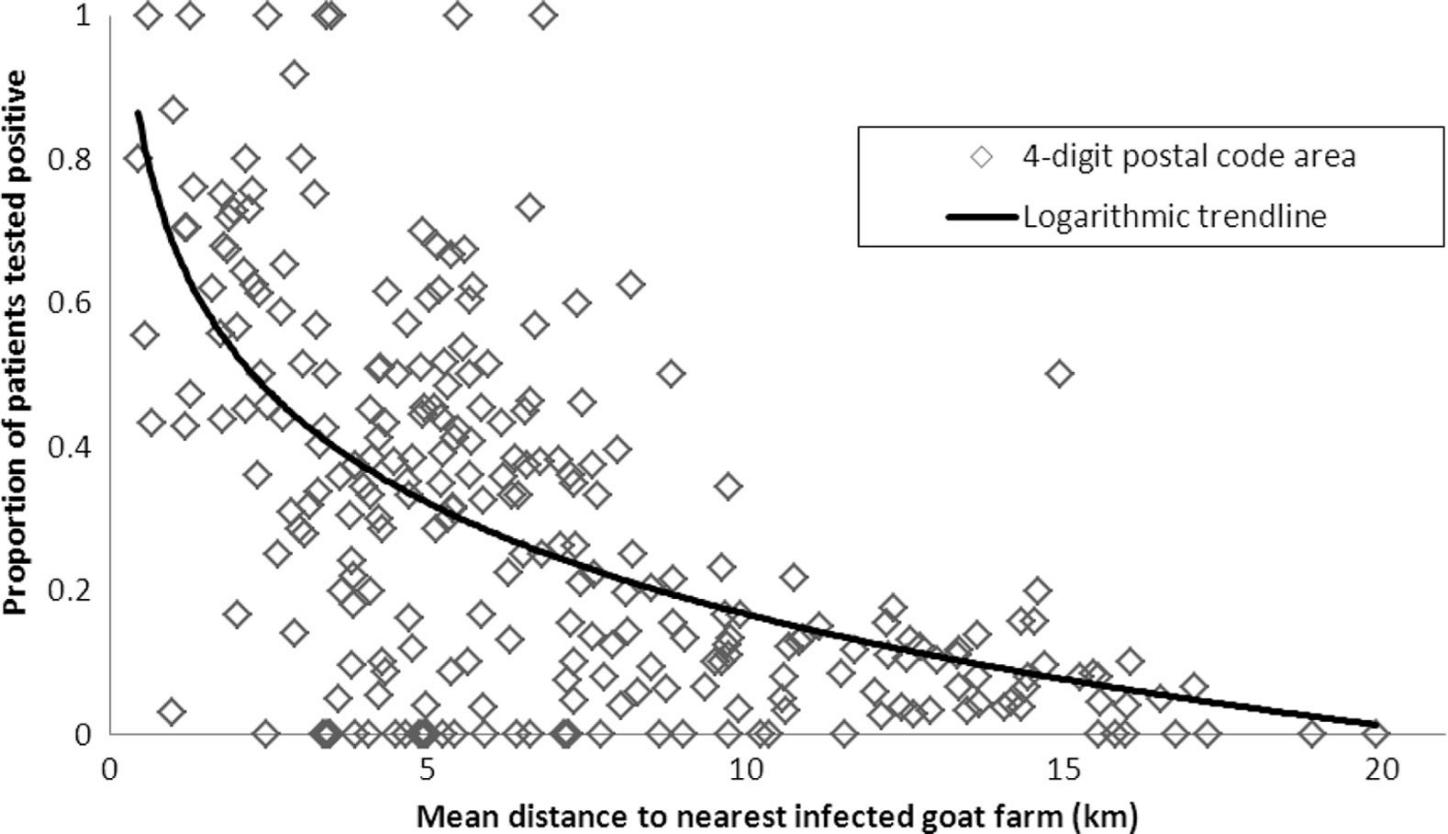
Proportion of patients testing positive for Q fever against mean distance from nearest infected dairy goat farm, per four-digit postal code area (*n*=262 postal codes areas).

Including distance from residential address to the nearest infected farm as a continuous variable rather than a categorical variable resulted in an OR of 0.79 (95% CI 0.78–0.80), that is, for a 1-km increase in distance, there is a 21% decrease in the odds of having a positive test result. The exponential decline in the proportion of patients testing positive with increasing mean distance from infected dairy goat farms for the 262 postal codes included in the study is shown graphically in Fig. 3. Areas with more positive test results compared to negative test results (proportion positive test results >0.5) are not seen at a mean distance of >8 km to the nearest farm.

Separate analyses with distance to bulk tank milk positive farms and distance to farms with confirmed abortion waves gave comparable results (data not shown). However, the logistic regression model with both types of infected dairy goat farms included was best in predicting the laboratory test outcome.

## Discussion

Published studies on human Q fever incidence and risk have used data on notified Q fever cases. In this article, we present a more comprehensive approach that accounts for differences in health-seeking and ‘testing behaviour’ during the epidemic. Some areas with many diagnostic tests performed had low numbers of positive test results and could be categorised as low risk. High proportions of positive test results were seen close to infected dairy goat farms.

Our study supports the previous research in the same area which links the risk of acquiring human Q fever with proximity to infected dairy goat farms (9, 15). It confirms that residential address is a good proxy for environmental exposure to *C. burnetii*. Environmental microbiology studies show a reduction in *C. burnetii* DNA content in aerosol samples from infected goat farms with increasing distance (16). The predominance of men, aged 20–60 years in the positive test results is in agreement with earlier studies (7, 17). The relatively low number of tests and positive test results in children may partly be explained by lower exposure, but it has been argued that children are less frequently symptomatic than adults following infection, and may have a milder disease and are therefore not tested (18). The sex difference in Q fever under the same exposure conditions for men and women has been explained by the protective effect of the 17-beta-oestradiol hormone in women (19).

During the epidemic, the link to goat farming has received substantial attention in the public media. Awareness of Q fever among patients and physicians can influence health-seeking behaviour and laboratory testing practices. Persons living close to goat farms may have sought medical care more rapidly than those who lived far from goat farms. The results of this study showed that indeed more persons were tested close to goat farms but the proportion of positive patients was also high in these areas. Low-risk areas were found further away from infected goat farms, where fewer persons were tested and where the proportion of positive tests was considerably lower. The number of patients testing positive was rapidly decreasing with increasing distance from infected goat farms, whereas the number of negatives was only slightly decreasing. In another study in the high-incidence area in the south of the Netherlands, stored sera that were collected during routine antenatal care were retrospectively analysed for antibodies against *C. burnetii* and these unbiased serological data confirm the importance of proximity to dairy goat farms (20).

We classified areas where fewer than five persons were tested as having ‘unknown risk’. These areas included an airport and industrial areas. The north-eastern part of the study area was close to another laboratory, and therefore it is likely that few serum samples were sent to the two regional laboratories that we selected for data collection in this study. Other possible explanations could be that no patients with symptoms presented at the physician, physicians did not refer patients for Q fever testing or there was no risk for Q fever in that area. Areas without an adequate number of patients tested for Q fever were not categorised as high- or low-risk areas to prevent misclassification. Data were collected from two regional laboratories with overlapping catchment areas. At the periphery of our study area, other laboratories would have played a role and total number of tests in those areas, even those with more than five tests, might have been underestimated. Therefore, the estimates of the proportion positive tests might be less robust at the periphery.

Most acute *C. burnetii* infections are asymptomatic, and this can affect the results, as asymptomatic infected patients would not have been included in the present study because we included only tests that were done for diagnostic purposes. However, criteria for requesting a diagnostic test could have changed with increasing awareness. As a consequence, the group of patients subjected to diagnostic testing widened from acute illness to for example, persistent fatigue.

During the study period, several control measures were implemented (8). Vaccination of goats started in April 2009; bulk tank milk monitoring in October 2009 with culling of pregnant goats on bulk tank milk positive farms in December 2009. After bulk milk tank monitoring started and locations of infected farms were communicated to the public, a slight increase in numbers of requested Q fever tests was observed (data not shown), which could have led to more Q fever case finding. Also, the culling of pregnant goats could have influenced the study results as the possible sources of exposure were removed. This could have weakened the relation between the risk for acute Q fever and distance to infected dairy goat farms. Furthermore, the exact infectious periods for the farms in the study are unknown.

The two regional laboratories used different testing methods for the detection of *C. burnetii* infections. The laboratory in Veldhoven mainly used CFT, and the laboratory in 's-Hertogenbosch used IFA. However, studies have shown that the different serological tests were reliable in detecting acute Q fever infections and perform equally well (13).

A limitation of the study is that the distance from residential address to the nearest infected farm was the only distance-related variable used. Work is on-going to develop a transmission model that allows for multiple sources and takes into account the number of animals on a farm and animal densities. The model will also take into account factors that play a role in the transmission from farm to humans, such as wind, particulate matter, vegetation patterns, and soil conditions around infected farms (21, 22). Furthermore, we assumed that persons became infected at their home, although infection might have occurred elsewhere. This might have weakened the association between location and infected farms because of non-differential misclassification. Nevertheless, we found a strong effect of distance to infected dairy goat farms.

In conclusion, by using laboratory test results, we identified high- and low-risk areas for Q fever in two study areas in the Netherlands. We used retrospective data, but in principle, data from laboratory information systems are available in real time and are potentially useful for effective implementation of public health control measures. While Q fever notification data with positive laboratory results and information on infected small ruminant farms are very useful to identify high-risk areas, the present study also shows the potential of using negative laboratory results to identify low-risk areas. Knowledge on the geographical distribution of risk makes it possible to implement veterinary and public health control measures in an efficient way by avoiding blanket coverage with uniform measures over a large area.

## References

[1] EFSA Panel on Animal Health and Welfare (1595). Scientific opinion on Q fever. EFSA J.

[2] Angelakis E, Raoult D (2010). Q fever. Vet Microbiol.

[3] Benenson AS, Tigertt WD (1956). Studies on Q fever in man. Trans Assoc Am Physicians.

[4] Maurin M, Raoult D (1999). Q fever. Clin Microbiol Rev.

[5] Schimmer B, Morroy G, Dijkstra F, Schneeberger PM, Weers-Pothoff G, Timen A (2008). Large ongoing Q fever outbreak in the south of The Netherlands, 2008. Euro Surveill.

[6] Schimmer B, Dijkstra F, Vellema P, Schneeberger PM, Hackert V, ter Schegget R (2009). Sustained intensive transmission of Q fever in the south of the Netherlands, 2009. Euro Surveill.

[7] Dijkstra F, van der Hoek W, Wijers N, Schimmer B, Rietveld A, Wijkmans CJ (2012). The 20 07–20 10 Q fever epidemic in The Netherlands: characteristics of notified acute Q fever patients and the association with dairy goat farming. FEMS Immunol Med Microbiol.

[8] Roest HI, Tilburg JJ, van der Hoek W, Vellema P, van Zijderveld FG, Klaassen CH (2011). The Q fever epidemic in The Netherlands: history, onset, response and reflection. Epidemiol Infect.

[9] Schimmer B, Ter Schegget R, Wegdam M, Zuchner L, de Bruin A, Schneeberger PM (2010). The use of a geographic information system to identify a dairy goat farm as the most likely source of an urban Q-fever outbreak. BMC Infect Dis.

[10] van der Hoek W, van de Kassteele J, Bom B, de Bruin A, Dijkstra F, Schimmer B (2012). Smooth incidence maps give valuable insight into Q fever outbreaks in the Netherlands. Geospat Health.

[11] van der Hoek W, Dijkstra F, Schimmer B, Schneeberger PM, Vellema P, Wijkmans C (2010). Q fever in the Netherlands: an update on the epidemiology and control measures. Euro Surveill.

[12] CBS Electronic Databank Statline http://statline.cbs.nl/StatWeb/dome/?LA=NL.

[13] Wegdam-Blans MC, Wielders CC, Meekelenkamp J, Korbeeck JM, Herremans T, Tjhie HT (2012). Evaluation of commonly used serological tests for detection of *Coxiella burnetii* antibodies in well-defined acute and follow-up sera. Clin Vaccine Immunol.

[14] Food and Consumer Product Safety Authority http://www.vwa.nl/onderwerpen/dierziekten/dossier/q-koorts/kaart-met-overzicht-van-besmette-bedrijven.

[15] Smit LA, van der Sman-de Beer F, Opstal-van Winden AW, Hooiveld M, Beekhuizen J, Wouters IM (2012). Q fever and pneumonia in an area with a high livestock density: a large population-based study. PLoS One.

[16] de Bruin A, Janse I, Koning M, de Heer L, van der Plaats RQ, van Leuken JP (2013). Detection of *Coxiella burnetii* DNA in the environment during and after a large Q fever epidemic in the Netherlands. J Appl Microbiol.

[17] Karagiannis I, Schimmer B, Van Lier A, Timen A, Schneeberger P, Van Rotterdam B (2009). Investigation of a Q fever outbreak in a rural area of The Netherlands. Epidemiol Infect.

[18] Maltezou HC, Raoult D (2002). Q fever in children. Lancet Infect Dis.

[19] Leone M, Honstettre A, Lepidi H, Capo C, Bayard F, Raoult D (2004). Effect of sex on *Coxiella burnetii* infection: protective role of 17beta-estradiol. J Infect Dis.

[20] van der Hoek W, Meekelenkamp JCE, Dijkstra F, Notermans DW, Bom B, Vellema P (2011). Proximity to goat farms and *Coxiella burnetii* seroprevalence among pregnant women. Emerg Infect Dis.

[21] van der Hoek W, Hunink J, Vellema P, Droogers P (2011). Q fever in The Netherlands: the role of local environmental conditions. Int J Environ Health Res.

[22] Reedijk M, van Leuken JPG, van der Hoek W (2013). Particulate matter strongly associated with human Q fever in The Netherlands: an ecological study. Epidemiol Infect.

